# Macroevolution of Flower Color Patterning: Biased Transition Rates and Correlated Evolution with Flower Size

**DOI:** 10.3389/fpls.2020.00945

**Published:** 2020-06-25

**Authors:** Matthew H. Koski

**Affiliations:** Department of Biological Sciences, Clemson University, Clemson, SC, United States

**Keywords:** correlated evolution, floral evolution, flower color, flower color pattern, hidden rates model, nectar guide, pollination, transition rate

## Abstract

Floral pigmentation patterns can both mediate plant-pollinator interactions and modify the abiotic environment of reproductive structures. To date, there have been no inquiries into the rate and directionality of macroevolutionary transitions between patterned and non-patterned petals despite their ecological importance and ubiquity across angiosperms. Petals in the Potentilleae tribe (Rosaceae) display color patterns in the ultraviolet (UV) and human-visible spectrum, or can be uniform in color (i.e., patternless). Using a phylogeny of Potentilleae, I test whether evolutionary transition rates between patterned and non-patterned petals are biased in either direction. I then examine whether UV and human-visible floral patterns are phylogenetically correlated and test the prediction that color patterns will evolve in concert with larger flowers if they function as guides to orient pollinators to floral rewards. I found that transition rates were biased toward petals that were uniform in color. Transition rates from patterned to uniformly colored petals were two and six times higher than the reverse for UV and human-visible pattern, respectively. The presence of UV and human-visible pattern evolved independently from one another. However, the evolution of human-visible pattern was associated with the evolution of larger flowers but the evolution of UV pattern was correlated with the evolution of smaller flowers. I posit that the transition bias toward non-patterned flowers may reflect developmental constraints on spatial regulation of pigments required to produce floral color patterning. The correlated evolution of larger flowers and human-visible pigmentation patterns support the hypothesis that nectar or pollen guides are more likely to evolve in larger-flowered species. This work provides insight into how transition rate bias and trait correlations can shape phylogenetic patterns of floral color pattern diversity.

## Introduction

**Figure 1:**
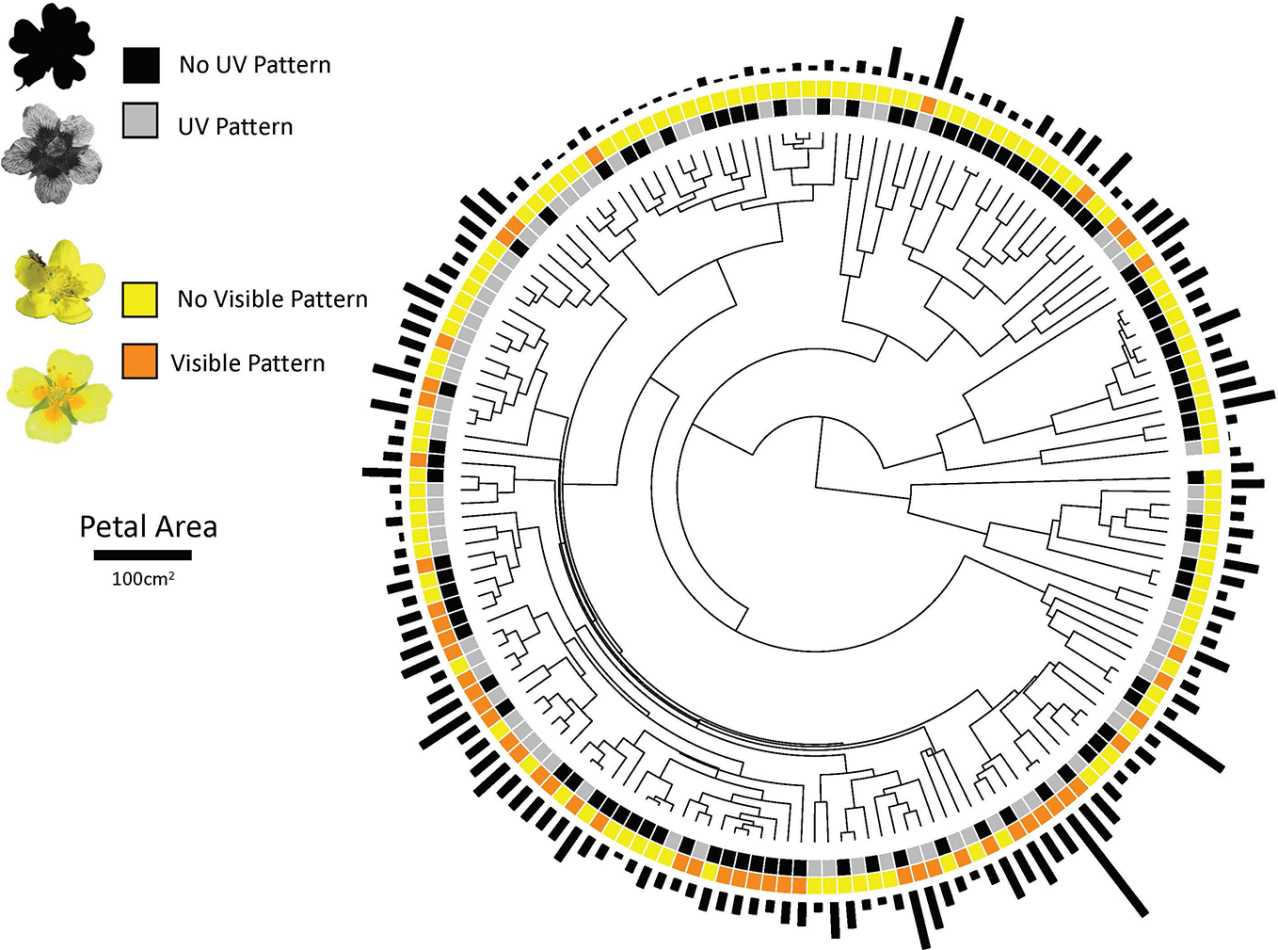
The phylogenetic distribution of ultraviolet and human-visible floral pigmentation patterns in and petal area in the Potentilleae tribe. Examples of species that lack UV pattern (*Potentilla evestita*), have UV pattern (*P. eriocarpa*), lack visible pattern (*Argentina anserina*), and have visible pattern (*P. erecta*) are provided in the key.

Petal color spots, patterns, and lines are common across angiosperms and are important for mediating plant-animal interactions. For example, color patterns on petals can enhance pollinator’s ability to detect flowers (Lehrer et al., 1995; Koski and Ashman, 2014), orient to floral rewards (e.g., Manning, 1956), and increase the likelihood of effective pollination (Hansen et al., 2012). Patterning can also discourage nectar robbers (Leonard et al., 2013) and function as deterrents to florivores (Gronquist et al., 2001). A particularly frequent floral color pattern is one whereby petal bases display different spectral signatures than petal apices, manifesting a bulleseye or target (e.g., Penny, 1983; Hempel de Ibarra and Vorobyev, 2009; Hempel de Ibarra et al., 2015; **Figure 1**). These “bull’s-eye” patterns are common in both the human-visible and ultraviolet (UV) spectrum (McCrea and Levy, 1983). While UV color patterns influence pollinator choice and behavior (e.g., Koski and Ashman, 2014; Peterson et al., 2015; Brock et al., 2016; Papiorek et al., 2016), there is also support for the role of UV patterns in protecting pollen from abiotic stress (Koski and Ashman, 2015a). Despite the ubiquity of color patterns and their myriad ecological roles in plant reproduction, we understand little about their evolutionary history.

Many studies have dissected the biochemistry and development of flower color patterning but none have evaluated their macroevolutionary dynamics. In fact, there are few studies on the tempo and directionality of macroevolution for ecologically relevant floral traits in general (however, see Smith and Goldberg, 2015; Wessinger et al., 2019 for pigment presence/absence; and Ree and Donoghue, 1999 for symmetry). Bull’s-eye pigmentation patterns can be manifested by the spatial regulation of pigments in multiple ways. First, pigments can accumulate at the base of petals but be absent at the tips (e.g., Harborne and Nash, 1984; Jorgensen, 1995). Alternatively, pigments can be restricted to petal tips but absent at the bases (e.g., Jorgensen, 1995). Thus, the absence of petal patterning could result from either the uniform production of a pigment across the petal or the loss of the pigment altogether. In either case, it is unknown whether evolutionary transitions between patterned and non-patterned flowers are biased in either direction. Determining transition rates in color patterns across a clade with both patterned and non-patterned flowers will give insight into whether there is asymmetry in evolutionary transitions between the character states. For example, an evaluation of the tempo and mode of floral color evolution across multiple clades revealed that gains in pigmentation were more common than losses (Smith and Goldberg, 2015).

The ecological functions of floral patterns, and correlations with other floral traits have the potential to influence their phylogenetic distributions. Experiments with natural and artificial flowers support that petal patterns influence pollinator alighting and orienting behaviors, but petal patterning may be a more salient cue for pollinators in larger flowers (Lawson and Rands, 2018). Bull’s-eye patterns increase the ability of pollinators to orient to the center of flowers (Manning, 1956; Free, 1970; Johnson and Dafni, 1998; Dinkel and Lunau, 2001; Lunau et al., 2009) and reduce handling time by pollinators (Waser and Price, 1985; Leonard and Papaj, 2011; de Jager et al., 2017). However, Koski and Ashman (2014) showed that in small radially symmetric flowers (~15 mm diam.), the presence of floral patterning failed to enhance the ability of small solitary bees and syrphid files to land on the center of flowers or orient to floral rewards after landing. Patterns that fail to enhance landing or orientation however may enhance contact between pollinators and anthers (see Dinkel and Lunau, 2001). Conversely, Kelber (1997) found that hawkmoths alighted to the periphery of large flower models lacking pattern (64 mm diam.). Likewise, Lunau et al. (2006) demonstrated that bumblebees approach petal peripheries in the absence of central color spots, again when the floral models were large (> 67 mm diam.). Together, these studies suggest that petal patterns may be more important in orienting pollinators in larger flowers. Additionally, a genetic algorithm that accounted for pollinator behavior predicted that larger flowers would be more likely to develop floral guides (Lawson and Rands, 2018). If pollinator-mediated selection drives the evolution of bull’s-eye patterns, we may expect a positive phylogenetic correlation between flower size and the presence of floral patterning.

Some surveys of petal color patterning in flowering plant communities support that patterning is more common in larger-flowered species. A survey of UV floral pattern in 300 Californian taxa found that the presence of pattern increased with flower size (Guldberg and Atsatt, 1975). This finding was corroborated in a broad survey of the Hawaiian flora (Jones et al., 1999). However, Dyer (1996) found that intermediate-sized flowers were more likely to possess UV-reflection in the Australian flora. A comparative lens using a group of species with and without petal pattern is one way to shed light on the hypothesis that patterns will be more likely to evolve in larger-flowered species.

In the Potentilleae, flowers can display human-visible (Asker, 1985; **Figure 1**) or UV bull’s-eye patterns (Koski and Ashman, 2016; **Figure 1**). UV petal patterning is caused by the production of various UV-absorbing flavonol compounds present at the base of petals but absent at the tips of petals (Harborne and Nash, 1984). Petals are often uniformly pigmented by carotenoids which do not strongly absorb UV (Goodwin, 1980). Thus, flowers that are uniformly UV-absorbing produce flavonols throughout the entire petal. Across nearly 200 Potentilleae species, roughly 50% are uniformly UV-absorbing while the other half are patterned (Koski and Ashman, 2016; **Figure 1**), making it an ideal group for macroevolutionary studies of floral color patterns. Human-visible bull’s-eye patterns are also present in Potentilleae due to dark yellow to orange pigmentation at the base of petals with brighter yellow petal apices (**Figure 1**). The biochemistry of visible patterning has not yet been illuminated in Potentilleae, however pigments underlying human-visible patterning are likely carotenoid compounds that are restricted to petal bases. Since UV and human-visible patterns are relevant to pollinator visual systems (Briscoe and Chittka, 2001), expression of both patterns which utilize different biochemical pathways could be functionally redundant with respect to mediating plant-pollinator interactions. If that is the case, visible patterns and UV patterns may display negative phylogenetic correlation such that visible patterns are more likely to evolve in species that uniformly absorb UV, and UV patterns would be more likely to evolve in flowers that lack visible pattern.

Using a phylogeny of Potentilleae, I characterize the presence and absence petal patterning in the UV and human-visible spectrum, as well as flower size to answer the following questions: 1) Are transitions between patterned and patternless flowers uniform or biased in one direction? 2) Are the evolution of UV and human-visible petal patterning correlated? 3) Are bull’s-eye patterns more likely to evolve in larger flowers?

## Methods

### System

Potentilleae is a globally distributed group of an estimated ~500 taxa. Flowers are all radially symmetrical, and the majority are yellow, though white and red/pink flowers have evolved. Flowers are highly generalized with respect to pollination, being pollinated by various fly species, solitary bees, and in some cases butterflies. One study revealed that both flies and solitary bees effectively transfer pollen (Koski and Ashman, 2015b). Harborne and Nash (1974) characterized the biochemical properties of flowers in 26 Potentilleae taxa; 16 with UV bull’s-eye patterns and 10 without. Various flavone, flavonone, and flavonol compounds were responsible for UV absorption in both patterned and non-patterned flowers. Carotenoids do not absorb UV, and have been identified as the yellow pigments in multiple *Potentilla* species (Goodwin, 1980). Basal petal pigmentation spots or bull’s-eye apparent in the human visible spectrum have not been characterized biochemically in Potentillae. However, they are often dark yellow to orange which is characteristic of carotenoid pigmentation (Goodwin, 1980). In one species with human-visible pattern (*Potentilla recta*), epidermal petal peels indicate that darker petal bases are caused by carotenoid pigmentation (**Supplemental Figure S1**).

### Scoring Petal Pattern and Flower Size

UV pattern and petal area were scored by photographing pressed flowers on an average of 3.2 herbarium specimens per 177 species (see Koski and Ashman, 2015). Petal area was scored as the area of one petal per flower in mm^2^. Species were considered patterned in the UV spectrum if a the average proportional petal area that absorbed UV was ≤95%. Two taxa were uniformly UV-reflecting but binned into the UV pattern character state. Seven taxa were 90% to 95% absorbing but were binned into the non-patterned character state. Petal pattern in the human-visible spectrum could not be scored from herbarium records because human-visible pigments often degrade in pressed flowers (Koski, pers. obs.). The presence or absence of human-visible petal patterns were obtained using images from online databases (iNaturalist, Calflora, GBIF, USDA Plants) or descriptions from online flora (e.g., Flora North America, Flora of China). Visible petal pattern was scored as “present” or “absent.” For 13 species, I was unable to obtain data on visible petal patterning from online sources. For any comparative model that contained human-visible pattern, the data set and phylogeny were truncated to 164 species that had data for all three traits scores; UV pattern, human-visible pattern, and petal size. For taxa that possessed both UV and human-visible petal patterning, I could not determine whether basal petal spots were congruent (i.e., completely overlapping) because UV images were obtained from herbarium specimens while human-visible pattern data was obtained from online images. Additionally, because human-visible pattern was obtained from standard human-visible images, the presence of “blue bull’s-eye’s” that may be perceived by pollinators (e.g., Verhoeven et al., 2018) but not humans, were not considered. Finally, I only considered petal patterning but not floral patterns caused by contrasting reproductive structures (e.g., Lunau, 2006).

### Phylogeny

For comparative analyses I used the time-calibrated Bayesian phylogeny of 183 Potentilleae generated by Koski and Ashman (2016) by combining two nuclear (ITS, ETS) and one chloroplast (trnLF) marker. For the current study, 200 phylogenies from the posterior distribution were trimmed to represent the species for which UV pattern, human-visible pattern, and petal size data could be obtained. All phylogenetic analyses were conducted on 200 phylogenies from the posterior distribution to account for phylogenetic uncertainty. For analyses that included UV pigmentation pattern and petal size, phylogenies were trimmed to 177 species. For analyses that included visible pigmentation pattern, phylogenies were trimmed to 164 species.

### Evolutionary Transition Rates Between Patterned and Patternless Flowers

To estimate transition rates between patterned and non-patterned flowers in both the UV and human-visible spectrum, I used hidden rate models (Beaulieu et al., 2013). Hidden rate models allow for variation in transition rates among lineages and account for the effects of unmeasured correlates of character states on transition rates. I used the hidden rate model for two primary reasons. First, for phylogenies that are large in taxonomic and geographic scope (e.g., the Potentilleae phylogeny used here samples from 5 genera with a global distribution), homogeneity in evolutionary rates across lineages is less likely (Beaulieu et al., 2013). Second, the hidden rate model may also account for the effects of unmeasured traits or environmental factors on evolution of the focal trait (Beaulieu et al., 2013).

I tested three transition rate models for each patterning trait: 1) a model with no hidden transition rates, 2) a model with one hidden rate allowing for “fast” and “slow” transitions, and 3) a model with two hidden rates allowing for “fast,” “medium,” and “slow” transitions (see Rivkin et al., 2016). Each of the six models (3 for UV pattern, 3 for human-visible pattern) were performed on 200 phylogenetic trees from the posterior distribution. For the model of UV patterning, 10 random starts were used per model optimization. However, for visible pattern, 5 random starts were used due to constraints on computational resources. I compared model fits between models 1 and 2, and models 2 and 3, to determine whether the addition of a hidden rate improved model fit. Model comparison was performed using a log-likelihood ratio tests. Average transition rates of the best-fit model across all 200 trees are presented. The marginal ancestral states were plotted on the Maximum Clade Credibility (MCC) tree generated from a corHMM model with 50 random starts.

### Testing for Correlated Evolution

To test whether human-visible pattern was more likely to evolve in flowers that lacked UV patterning, I used Pagel’s Test for correlated evolution between visible patterning and the absence of UV patterning (Pagel, 1994). Pagel (1994) test compares model fit parameters between an evolutionary model whereby transitions in one binary trait depend on another binary trait, and a model whereby each trait evolves independently. Using the corDISC function (corHMM package), I fit a model where the transitions between visible pattern and patternless flowers were dependent on transitions between UV-patterned and paternless flowers permitting different transition rates among characters (“ARD” option). I calculated the average log likelihood across all 200 posterior trees and compared it to the summed the log likelihood values from the independent models for UV pattern and human-visible pattern with zero hidden rates using a likelihood ratio test (Rivkin et al., 2016). If the log-likelihood was significantly higher for the dependent model compared to the independent model, I concluded that the evolution of human-visible pattern was correlated with the evolution of UV pattern.

To test whether the evolution of patterned flowers (UV- and human-visible) were associated with the evolution of larger-flowered species, I used generalized estimating equations (Paradis and Claude, 2002; GEE; compar.gee function, “ape” package). Specifically, I modeled the presence (1) and absence (0) of pattern as a function of petal size with a binomial distribution, a logit link function, and the phylogeny with branch lengths transformed to 1 as the correlation structure. I used separate models for the presence/absence of human-visible patterning and the presence/absence of UV patterning. Petal area was log-transformed to achieve normality. GEE models were run on the 200 posterior trees, and model parameters were averaged. The model for human-visible patterning failed to converge on 8 of the 200 posterior trees (4%), thus I report the average parameter values from models using 192 trees. When there was a significant phylogenetic relationship between petal size and petal patterning based on the GEE results, I generated PIC values for each trait using the MCC phylogeny to visualize the relationship.

## Results

### Floral Color Pattern

Of 177 species in the Potentilleae tribe examined in this study, 48% possessed UV floral patterning (n = 85) while 93 were uniformly UV-absorbing. Fewer species (n = 50 of 164 with data available on visible patterning; 30.5%) had visible petal patterning while the remaining were uniform in color. For all species with data available for both UV and visible patterning (n = 164), 23 possessed both UV and visible pattern, 52 had UV pattern but lacked visible pattern, 27 were visibly patterned but lacked UV pattern, and 62 displayed neither UV nor visible patterning (**Figure 1**). Thus, the majority of species (~63%) display petal patterning in the visible spectrum, UV spectrum or both.

### Transition Rates—UV Patterning

**Figure 2:**
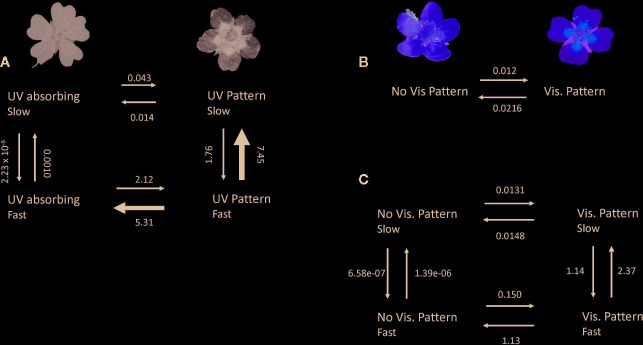
Evolutionary transition rates among patterned and patternless flowers in **(A)** the UV spectrum given the best fit model of a single hidden rate, **(B)** the human-visible spectrum given a model with no hidden rates, and **(C)** the human visible spectrum given a model with a single hidden rate. The single hidden rate model for visible pattern is a marginally better fit than the model without a hidden rate (*P* = 0.054). Arrow widths are proportional to rates. Rates are presented in events per million years.

For evolutionary transition rates between UV-patterned and patternless species, a model with one hidden rate was supported (**Table 1**). That is, transition rates between patterned and non-patterned flowers in the UV spectrum varied across the phylogeny between two rates (**Supplemental Figure S1**). The transition rate from uniformly UV-absorbing to UV-patterned flowers was 2.163 while the reverse was 5.323 (**Figure 2A**). Thus, transitions toward uniform UV absorption were about 150% as frequent as the reverse. Transition rates between UV pattern states (patterned fast and patterned slow) were however much higher than transition rates among the two patternless states (9.21 vs 0.001; **Figure 2A**).

**Table 1:** Model fit comparisons of evolutionary transition rates between patterned and non-patterned petals.

	Number of parameters	Log Likelihood	AIC	χ^2^	P
**UV color pattern**					
No hidden rates	2	−115.109	234.24		
One hidden rate	8	−107.898	231.79	14.422	0.020
Two hidden rates	14	−106.759	241.51	2.278	0.892
**Visible color pattern**					
No hidden rates	2	−91.4	186.81		
One hidden rate	8	−85.22	186.45	12.36	0.054
Two hidden rates	14	−84.726	197.45	0.988	0.986

The model with no hidden rates assumes that rates do not vary across the phylogeny. The “one hidden rate” model assumes that two rates can vary across the tree and the “two hidden rates” model assumes that three rates can vary across the tree. Chi-square values were generated from log-likelihoods between the more complex model against the simpler model (e.g., two rates vs. one rate) and P values were calculated using a log-likelihood ratio test. All models were run on 200 posterior trees to account for phylogenetic uncertainty and the average log likelihood values were compared.

Ancestral state reconstruction of characters and transition rates show that transitions rates vary across the phylogeny, but within given clades the most likely rate is largely consistent (**Supplemental Figure S1**). For example, uniform UV absorption (slow) dominated in the *Drymocallis* and Alba clades but was rare elsewhere in the phylogeny (**Supplemental Figure S2**). Uniform UV absorption (fast) was frequent in the Ivesiod clade and within some clades within the large *Potentilla* group. UV patterning (slow) was common in numerous clades within the *Potentilla* group and the Reptans clade (**Supplemental Figure S2**). The presence of UV pattern is the most likely character state for the ancestor of the diverse Potentilla group (> 75% likelihood; **Supplemental Figure S2**). UV pattern (fast) is an infrequent ancestral state because transitions away from this state are the most frequent (**Supplemental Figure S2**; **Figure 2A**).

### Transition Rates—Visible Patterning

For human-visible patterning, a model with one hidden rate was supported over a model without hidden rates (**Table 1**), though the likelihood-ratio test was only marginally significant (P = 0.054). Therefore, I report results of both the single- and 2-rate model. Given a single rate model (no hidden rates), transitions from patterned to non-patterned flowers were about 80% more frequent than the reverse (0.0216 vs. 0.012; **Figure 2B**). However, under a two-rate model, transitions from patterned to non-patterned flowers were about 6 times as frequent as the reverse (1.145 vs. 0.163; **Figure 2C**). In the model with one hidden rate, transitions among patterned states were again much higher than transitions among non-patterned states (3.51 vs. 0.00001; **Figure 2C**).

Ancestral states reconstruction for the model without a hidden rate for visible pattern shows that the presence of visible pattern dominated in the *Potentilla* group but the absence of visible pattern dominated in all clades outside of this group (**Supplemental Figure S2**). There is support for a visibly patterned common ancestor of *Potentilla* clade under models with or without a hidden rate (**Supplemental Figure S3**, **S4**).

### Correlated Evolution

Independent models of evolution for UV pattern and visible pattern were better supported than a model of dependent evolution (**Table 2**). That is, UV and visible color patterns evolved independently from one another. This is highlighted by the fact that visible color patterns were not more likely to occur in species that lack UV patterns. Of the 50 taxa that were visibly patterned, 27 (54%) were UV-patterned and 23 (46%) were uniformly UV-absorbing.

**Table 2:** Pagel (1994) test of correlated evolution for UV- and human-visible petal patterning in Potentilleae.

	Log Likelihood	χ^2^	*P*
Independent	−206.509		
Dependent	−203.678	5.662	0.226

The dependent model assumes that UV- and human-visible pattern evolve independently and the log-likelihood is the sum of log likelihoods from the single rate visible and UV pattern model (see **Table 1**). The dependent model assumes that the evolution of human visible patterning depends on the presence/absence of UV patterning. Log likelihoods were generated from running each model on 200 random posterior trees to account for phylogenetic uncertainty.

**Figure 3:**
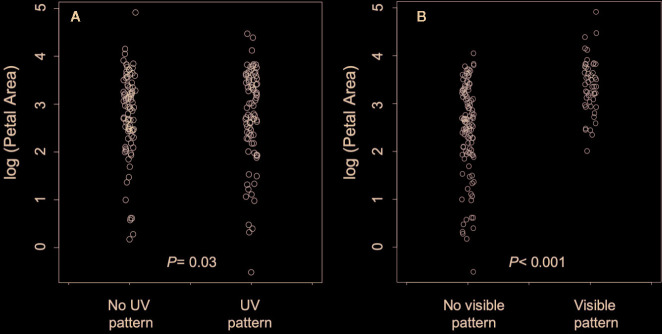
**(A)** The relationship between petal area and the presence/absence of UV pigmentation pattern. **(B)** The relationship between petal area and the presence/absence of human-visible pigmentation pattern. *P* values in each panel are from phylogenetically controlled generalized estimating equations testing the association between the presence of pattern and flower size.

Contrary to the prediction that floral patterns will evolve in concert with larger flowers, the presence of UV pattern was associated with smaller flowers (parameter estimate = −0.409 ± 0.16 SE; T= −2.73, df P = 28.1, *P* = 0.03; **Figure 3A**). On the other hand, the presence of human-visible patterning evolved in concert with the evolution of larger flowers as predicted (Parameter Estimate= 1.570 ± 0.33 SE; T= 4.75, df P = 27.6, *P* = 0.0004, **Figure 3B**). Thus, expectations were met for human-visible, but not UV pattern.

## Discussion

Comparative work in the Potentilleae tribe, a group that varies in the presence of petal color patterns, supports that evolution is biased toward the absence of floral patterning for both patterns caused by human-visible pigments and UV-absorbing pigments. Elevated rates of evolution toward non-patterned flowers support that evolution of color patterning is likely more developmentally complex than uniform production of pigments across the entire petal. Visible color patterning was not more likely to evolve in flowers that lacked UV petal patterning, and UV patterning was not more likely to evolve in flowers that lacked visible patterning, suggesting that visible and UV patterning may not be functionally redundant. Visible petal patterns evolved in concert with larger flowers, providing support for the hypothesis that “nectar guides” may be more likely to evolve in larger flowers. Conversely, UV patterns evolved in concert with smaller flowers suggesting that either UV patterns are not pollinator-orienting cues, or that smaller flowers evolve UV patterns to increase pollinator attraction from a distance. This study sheds light on the evolutionary history of petal patterning and sets the stage for studies examining the development and functional roles of floral color patterns in the Potentilleae tribe.

### Biased Transition Rates Toward Patternless Flowers

Assessing the results of transition rate analyses for both UV- and human-visible pattern provides a consistent trend of asymmetric transitions toward color uniformity. Biased evolutionary transitions toward uniform coloration may have developmental or ecological underpinnings. First, evolving pattern often requires precise regulation of the domain of expression of pigments (e.g., *via* alterations to MYB transcription factor expression or their binding sites; Mol et al., 1998; Quattrocchio et al., 2006; Jiang and Rausher, 2018; Ding et al., 2020). Thus, expression of uniform petal coloration may require fewer modifications in fewer structural or regulatory elements of pigmentation pathways compared to the precise spatial regulation of pigment production required for the evolution of color patterning. Additionally, color patterning could be easily lost in a lineage if it does not have a strong selective advantage over uniform coloration. In some systems for instance, selection for increased an increased UV-absorbing area on petals can be strong—Koski and Ashman (2015a) showed that elevated pigmentation protects pollen from UV stress and that across species in the Potentilleae those with ranges in higher-UV environments have elevated pigmentation (Koski and Ashman, 2016).

Past work has characterized the absence of pigmentation as a “loss” and the presence of pigmentation a “gain” (Zufall and Rausher, 2004; Smith and Goldberg, 2015). Treating UV color patterns in a similar binary manner, however, is difficult. The evolution of a uniformly UV-absorbing flower from a UV-patterned flower could be categorized as a gain in pigmentation (pigments are produced at the apex of the petal whereas they were not previously produced in an ancestor). Alternatively, this transition could be viewed as a loss of regulatory ability to restrict pigment production to the bases of petals. Moreover, for either character state, the UV-absorbing pigment is being produced, so the pigment itself is neither lost nor gained. Indeed others have cautioned against categorizing the evolution of a given character state as a loss or gain (Stern and Orgogozo, 2008). Regardless, the biased transition rate toward uniform UV absorption suggests that once petals produce UV-absorbing pigments uniformly across petals, evolving mechanisms to restrict expression to petal bases is rare.

Basal petal spots or patterns in the human-visible spectrum could also be considered a gain or a loss depending on the mechanism by which pigmentation patterns arise. While speculative, the presence of human-visible pigmentation pattern may be underlain by the production of a novel carotenoid that is absent in flowers that lack pattern. If this is the case, then patterning could be considered a gain in pigmentation. Thus, the biased transition rate toward the lack of pigmentation patterns in the human-visible spectrum would suggest that losses are more common than gains which differs from a comparative study on the presence or absence of anthocyanin pigmentation in three groups (Smith and Goldberg, 2015). If evolution toward uniform coloration is caused by the loss of a pigment, then this study is consistent with Dollo’s Law (Gould, 1970; Bull and Charnov, 1985) that suggests losses of complex characters are irreversible. The irreversibility of trait loss is expected due to the statistical unlikelihood of re-evolving the exact ancestral phenotype after its loss. For both UV- and human-visible pattern, biochemical studies will be a necessary first step to uncover the developmental basis of petal patterning in Potentilleae. Additionally, pinpointing the ancestral character states of Potentllieae, and estimating the number of evolutionary transitions between states will be important for fully assessing petal patterns in the context of Dollo’s Law.

Biased transitions toward color uniformity may have ecological implications with respect to plant-pollinator interactions as well as protection of pollen and ovules from abiotic stress. First, the loss of petal pattern could change the efficiency of foraging by pollinating insects (e.g., Dinkel and Lunau, 2001; Lunau et al., 2006), and if patterns are important to some but not all pollinators, evolutionary transitions to color uniformity could be accompanied by a transition in dominant pollinators. A thorough investigation of pollinator importance across taxa in Potentilleae would be important to examine the role of pollinators in contributing to evolutionary transitions in petal pattern (e.g., Smith, 2010). Larger UV-absorbing areas on petals are associated with increased protection of pollen from UV stress (Koski and Ashman, 2015a), and the evolution of uniform UV absorption tends to be associated with geographic shifts into areas of elevated ambient UV-B irradiance (Koski and Ashman, 2016). Whether evolutionary shifts toward habitats with higher UV-B irradiance are also directional would shed light on how habitat shifts could underlie the biased transition rates.

Some caveats must be considered when interpreting the transition rates reported from the hidden-rates model. First, estimates of species diversity in the Potentilleae are somewhere over 500 species with five to six genera [Angiosperm Phylogeny Group; Steven (2017)]. While the phylogeny sampled 5 genera spanning a broad geographic range, missing taxa could skew results. Second, the transition rates obtained could be confounded by state-dependent diversification (Beaulieu and O’Meara, 2016). For instance, biased transition rates toward a given state could be driven by increased diversification rates of lineages with the given state (Ng and Smith, 2014). While this could be the case for human-visible patterning (~70% of taxa sampled were uniformly colored), I do not expect that biased rates toward uniform UV coloration are driven by an abundance of non-UV-patterned species in the phylogeny of Potentilleae (~50 were uniformly UV-absorbing while half were UV-patterned). Regardless, increased sampling in Potentilleae, increased phylogenetic resolution, and employment of state-dependent models of diversification would help to strengthen the understanding of macroevolutionary transitions in petal color patterning in this group.

### Phylogenetic Trait Correlations With the Evolution of Floral Patterning

I predicted that human-visible and UV pattern would display negative phylogenetic covariance if each type of petal pattern were functionally redundant for enhancing pollinator attraction and orientation. That is, visible patterns should evolve when flowers are uniformly absorbing and UV patterns should evolve when flowers are visibly uniform in color. The fact that this is not the case in Potentilleae suggests that human-visible and UV patterning are unlikely to be functionally redundant as pollinator-orienting cues. Indeed, UV pigmentation protects pollen from UV damage in addition to increasing attractiveness to pollinators (Koski and Ashman, 2014; Koski and Ashman, 2015b), but the ecological role of visible patterning is unknown in this group. From a biochemical standpoint, the lack of covariance between UV- and human-visible patterning may not be surprising given that UV absorption is manifested through flavonoids while darker-orangish petal bases are likely caused by carotenoids. Because pigments underlying human-visible and UV patterning are likely produced by independent pigment pathways, the evolution of human-visible and UV patterning may thus be independent.

The evolution of patterning in the human-visible spectrum and UV spectrum differ in their relationship with the evolution of flower size. While previous work has suggested that across diverse plant communities, the presence UV patterning is associated with larger flowers (Guldberg and Atsatt, 1975), this is not the case when conducing a phylogenetically controlled analysis in Potentilleae. In fact, the evolution of larger flowers was associated with the evolution of uniform UV absorption, contrary to the expectation if UV patterns were pollinator-orienting cues. However, one cannot rule out that the observed pattern could be explained by pollinator mediated selection. If smaller flowers are less attractive to pollinators than larger flowers (Johnson et al., 1995; Conner and Rush, 1996) they may be under selection to evolve additional visual signals to attract pollinators, like UV-reflective patterns (e.g., Hirota et al., 2019). Additionally, if smaller flowers are pollinated by small insects with low-resolution compound eyes, floral guides may still be important for orienting pollinators once they are very close to flowers and are able to distinguish the patterns (Hempel de Ibarra et al., 2015).

There was strong evidence for the correlated evolution between larger flowers and the presence of human-visible color patterning, supporting the prediction that flower color patterns may act as important cues to orient pollinators in larger flowers. For instance in large flowers, central petal spots increase pollinator’s ability to alight to the center of flowers (Kelber, 1997; Lunau et al., 2006). This phylogenetic pattern has not yet been documented in other systems, but corroborates two studies showing an increased likelihood of petal color patterning in larger-flowered species in two communities (Guldberg and Atsatt, 1975; Jones et al., 1999) as well as a genetic algorithm predicted that larger flowers are more likely to evolve floral guides (Lawson and Rands, 2018). To my knowledge, behavioral studies that evaluate the efficacy of bull’s-eye patterns as attracting and orienting cues for pollinators in flowers of varying size have not been done but will be important for determining the potential driver of this phylogenetic pattern.

## Conclusions

Flowering plants display enormous variation in petal color patterns, and numerous studies have evaluated their ecological significance and development. This is the first study to draw on these ecological and developmental studies to assess how they may shape macroevolutionary patterns and processes for floral color patterns. Developmental constraints may slow the rate of evolution of petal patterning from uniformly colored ancestors leading to bias in evolutionary transition rates. Additionally, intrinsic genetic or ecologically driven correlations between flower size and color patterns are important for shaping the phylogenetic distribution of color patterning. Whether the efficacy of petal patterns as pollinator-orienting cues depends on flower size, and dissection the biochemical and genetic underpinnings of pattern evolution in Potentilleae are underway.

## Data Availability Statement

The data sets presented in this study can be found in online repositories. The names of the repository/repositories and accession number(s) can be found below: https://datadryad.org/stash/share/8EpJTtK7Q3639i44NQy4wC6gAwx2JF9qtDgfQhICMn0.

## Author Contributions

MK conceived of the study, collected and analyzed the data, and wrote the manuscript.

## Conflict of Interest

The author declares that the research was conducted in the absence of any commercial or financial relationships that could be construed as a potential conflict of interest.
