# Supplementary material for: Macroevolution of Flower Color Patterning: Biased Transition Rates and Correlated Evolution with Flower Size
Source: Front Plant Sci. 2020 Jun 25;11:945. doi: 10.3389/fpls.2020.00945 (PMC7344184; doi:10.3389/fpls.2020.00945)
Supplement: Supplementary file 1 [file DataSheet_1.pdf]

Supplementary Materials for Koski 2019, "Macroevolution of flower color patterning: biased transition rates and correlated evolution with flower size"

Figure S1: Epidermal petal peels of *Potentilla recta*, a species with pale yellow flowers and darker yellow petal bases. **A** and **B** show cells at the apex of petals with carotenoids in plastids. Panel **B** shows a side-view of conical cells. Panels **C** and **D** display basal petal epidermal cells with carotenoids in plastids with D giving the side-view of the conical epidermal cells. Carotenoids are bound in plastids so cells are not uniformly colored by carotenoids. Alternatively, anthocyanins are vacuolar and provide more-or-less uniform coloration across cells (Ng and Smith 2016). The petals peels support carotenoids as the pigment giving rise to petal patterning in at least one species.

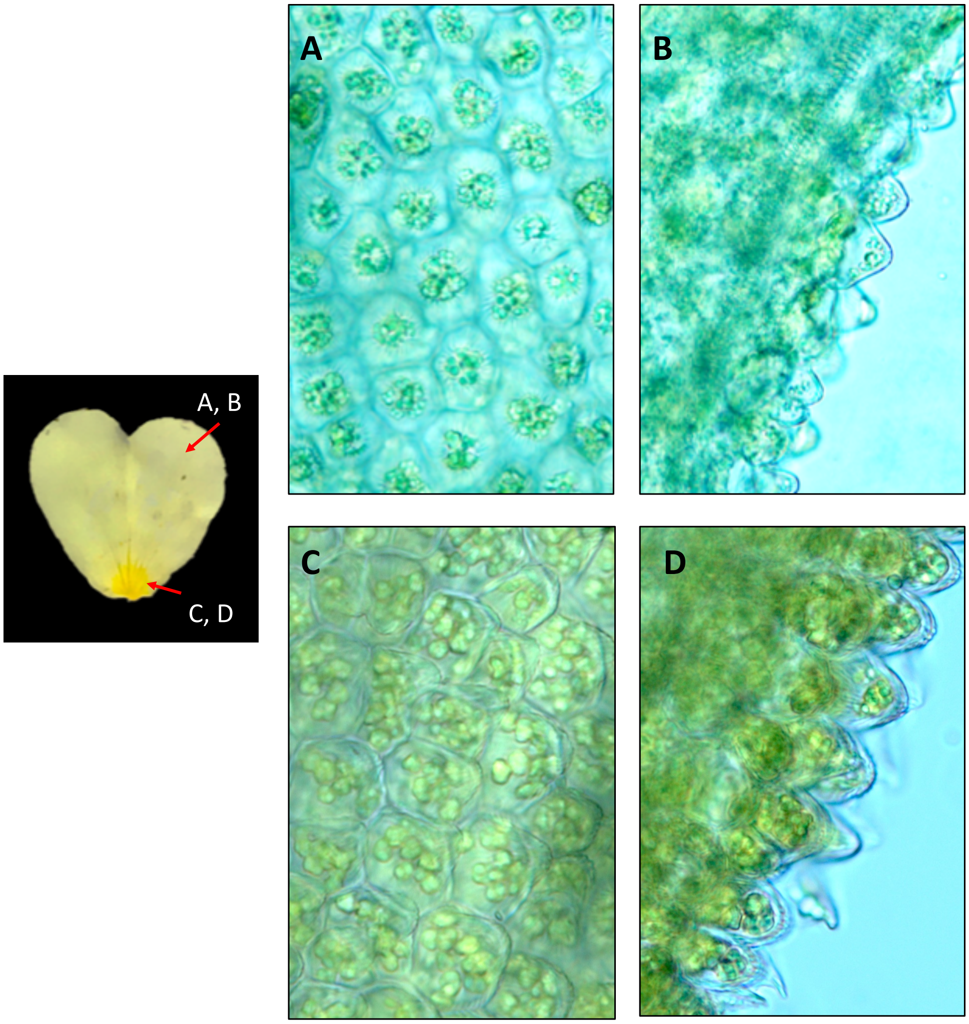

Figure S2: Marginal ancestral state reconstruction of UV pattern presence/absence for 177 species in the Potentilleae Tribe. The ancestral nodes of major clades are noted. The best fit hidden rate model included one hidden rate so transition rates vary between ‘fast’ and ‘slow’ across the phylogeny.

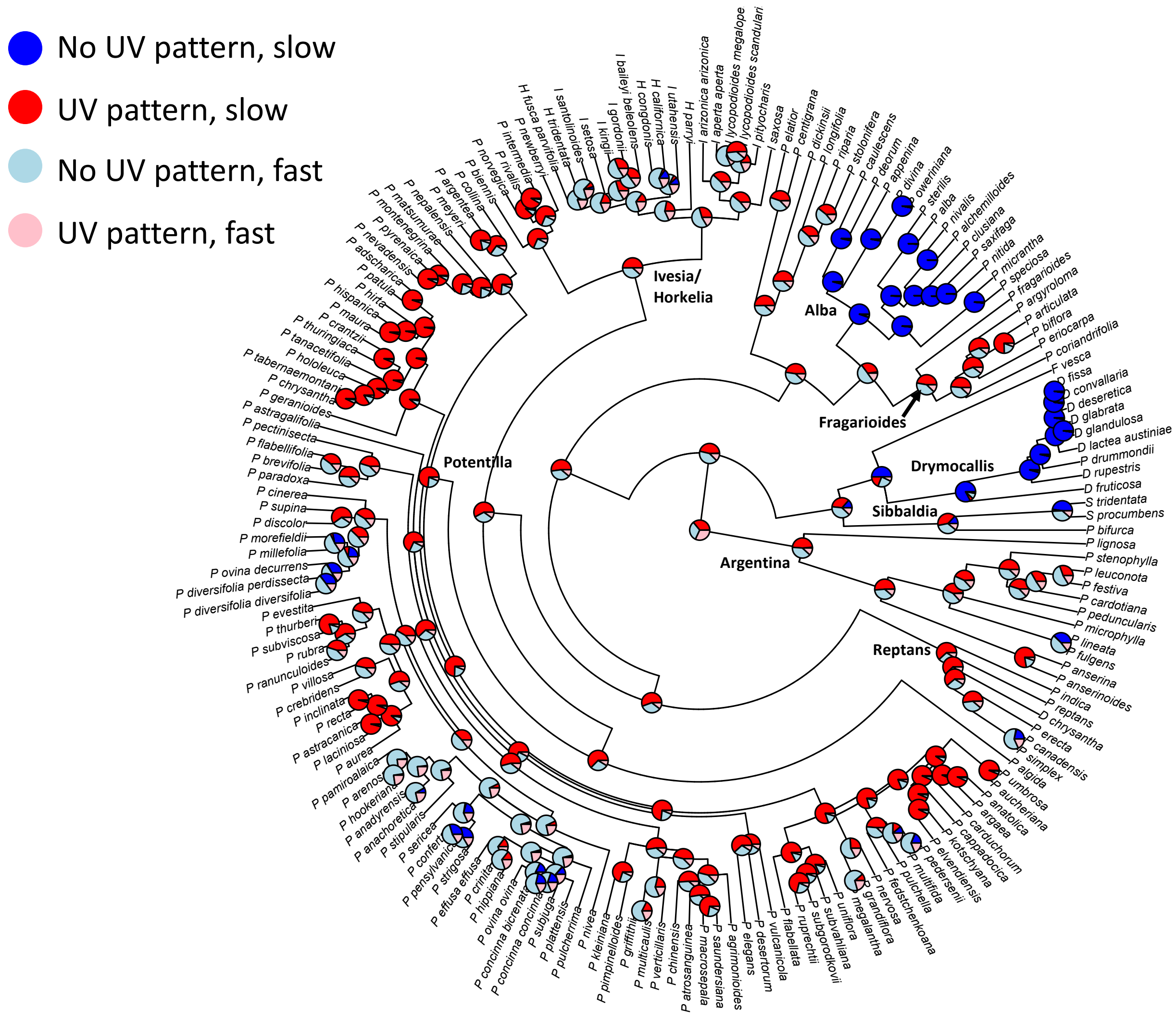

Figure S3: Marginal ancestral state reconstruction of human-visible pattern presence/absence for 164 species in the Potentilleae Tribe for an evolutionary model with no hidden rates. The ancestral nodes of major clades are noted.

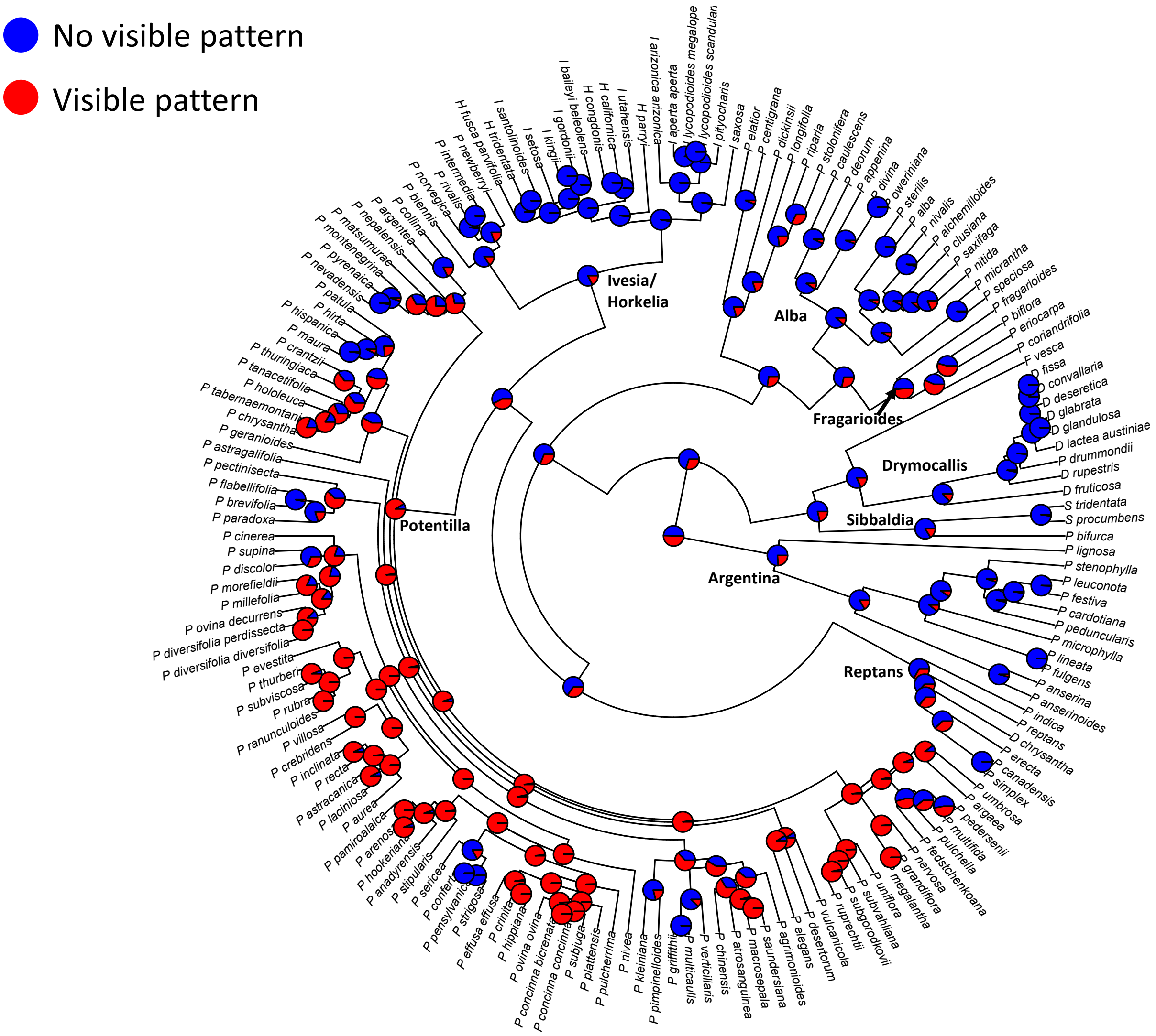

Figure S4: Marginal ancestral state reconstruction of human-visible pattern presence/absence for 164 species in the Potentilleae Tribe for an evolutionary model with one hidden rate. The ancestral nodes of major clades are noted. The model with one hidden rate provided a better fit than the model without hidden rates (Fig S2), but the increased quality of the fit was modest (Table 2 in main body of manuscript).

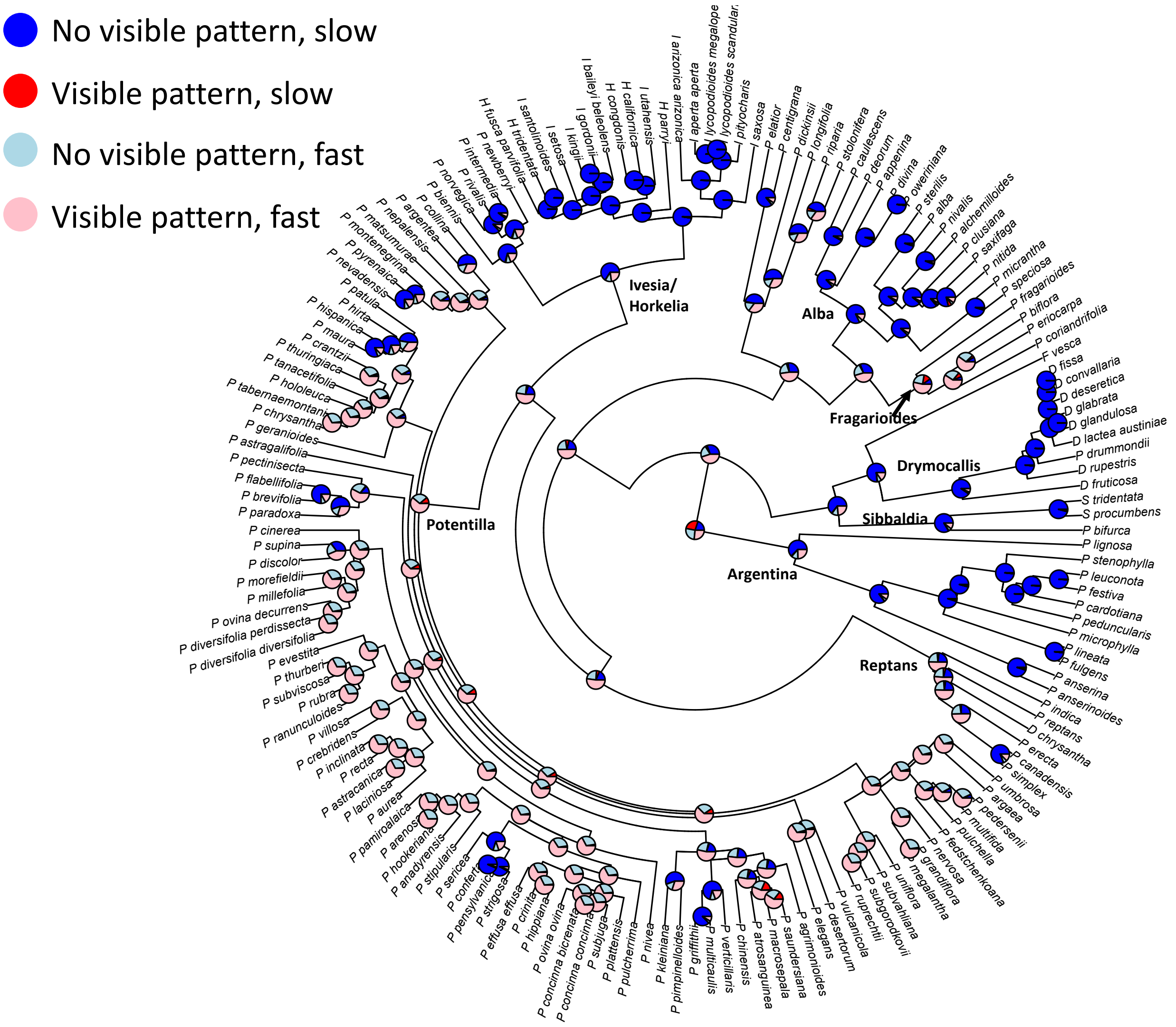

References:

Ng, J., and Smith, S. D. (2016). Widespread flower color convergence in Solanaceae via alternate biochemical pathways. *New Phytologist* 209, 407–417. doi:10.1111/nph.13576.
